# Organic acids for control of *Salmonella* in different feed materials

**DOI:** 10.1186/1746-6148-9-81

**Published:** 2013-04-18

**Authors:** Sevinc Koyuncu, Mats Gunnar Andersson, Charlotta Löfström, Panagiotis N Skandamis, Antonia Gounadaki, Jürgen Zentek, Per Häggblom

**Affiliations:** 1 Department of Chemistry, Environment and Feed hygiene, National Veterinary Institute (SVA), Uppsala, Sweden; 2 National Food Institute, Technical University of Denmark (DTU), Søborg, Denmark; 3 Department of Food Science and Technology, Agricultural University of Athens (AUA), Athens, Greece; 4 Institute of Animal nutrition, Freie Universität Berlin (FUB), Berlin, Germany

**Keywords:** *Salmonella*, Feed materials, Organic acid, Survival analysis, Temperature effect, Acid tolerance

## Abstract

**Background:**

*Salmonella* control in animal feed is important in order to protect animal and public health. Organic acids is one of the control measures used for treatment of *Salmonella* contaminated feed or feed ingredients. In the present study, the efficacy of formic acid (FA) and different blends of FA, propionic acid (PA) and sodium formate (SF) was investigated. Four *Salmonella* strains isolated from feed were assayed for their acid tolerance. Also, the effect of lower temperatures (5°C and 15°C) compared to room temperature was investigated in rape seed and soybean meal.

**Results:**

The efficacy of acid treatments varied significantly between different feed materials. The strongest reduction was seen in pelleted and compound mash feed (2.5 log_10_ reduction) followed by rapeseed meal (1 log_10_ reduction) after 5 days exposure. However, in soybean meal the acid effects were limited (less than 0.5 log_10_ reduction) even after several weeks’ exposure. In all experiments the survival curves showed a concave shape, with a fast initial death phase followed by reduction at a slower rate during the remaining time of the experiment.

No difference in *Salmonella* reduction was observed between FA and a blend of FA and PA, whereas a commercial blend of FA and SF (Amasil) was slightly more efficacious (0.5-1 log_10_ reduction) than a blend of FA and PA (Luprocid) in compound mash feed. The *Salmonella* Infantis strain was found to be the most acid tolerant strain followed by*, S.* Putten*, S.* Senftenberg and *S.* Typhimurium*.* The tolerance of the *S.* Infantis strain compared with the *S.* Typhimurium strain was statistically significant (p<0.05). The lethal effect of FA on the *S.* Typhimurium strain and the *S.* Infantis strain was lower at 5°C and 15°C compared to room temperatures.

**Conclusions:**

Acid treatment of *Salmonella* in feed is a matter of reducing the number of viable bacterial cells rather than eliminating the organism. Recommendations on the use of acids for controlling *Salmonella* in feed should take into account the relative efficacy of acid treatment in different feed materials, the variation in acid tolerance between different *Salmonella* strains, and the treatment temperature.

## Background

The importance of animal feed as source for *Salmonella* dissemination has been highlighted by EFSA, particularly in countries with a low prevalence of *Salmonella* in primary production [1]. Animal feeds are at risk of *Salmonella* contamination at several stages in the feed chain starting with the production of ingredients. Different factors are known to influence the risk of introducing *Salmonella* into animal feed, including contaminated ingredients, contaminated feed mill environments causing re-infection of the feed, wild birds and rodents [2].

According to European feed legislation, Regulation (EC) No. 183/2005, feed business operators should apply HACCP principles and good hygiene practice/good manufacturing procedures (GHP/GMP) at each stage of the feed chain in order to secure safe feed. Several technical procedures are available for decontamination of *Salmonella* contaminated feed or feed ingredients, such as heat treatment and treatment by chemicals [3,4]. The use of organic acids varies between different countries depending on differences in legislation or other factors. In some countries, acids are used for decontamination of *Salmonella* in feed ingredients prior to heat treatment, while in other countries acids are primarily added to finished feed. One aspect that is often highlighted is that acids can provide some residual protection against recontamination compared to a heat treated product. Some acids may also prevent intestinal colonisation of *Salmonella* in animals [5].

Short-chain organic acids have been found to be effective against *Salmonella* in feed [3]. It has also been suggested that blends of acids may be more efficacious than single acids for reduction in *Salmonella*[5]. Commonly used acids are FA and mixtures of FA and PA, often in a ratio of 80%:20%.

In several studies, the effects of different combinations of acids including commercial mixtures on *Salmonella* in artificially contaminated feed materials were investigated [6-8]. It is well known that the efficacy of organic acids varies widely with the type of acid or blend of acids used, the physical nature of the acid, the inclusion rate, whether the products are present as free acids or salts and the level of *Salmonella* in the commodity [1]. The situation is well illustrated by Ha *et al.* that reported an approximately 1 log_10_ reduction with buffered propionic acid at an inclusion rate of 1% in poultry mash after 7 days of exposure, while Rouse *et al.* on the other hand obtained an almost 3 log_10_ reduction in *Salmonella* in poultry mash after 24 hours (h) exposure to a propionic acid-based chemical [6,8].

No studies seem to be published on acid treatments at temperatures below room temperature in feed ingredients. In addition, commercial suppliers rarely present data on the decontamination effects at temperatures other than room temperature. Cherrington *et al*. (1992) studied the effect of acetic and lactic acid and BioAdd at elevated temperatures in presence of blood, milk and serum for bactericidal activity against *S.* Kedougou [9]. The activity of the acids increased with temperature and time of incubation.

The temperature in the greater part of the year in the Nordic countries is low and for that reason, there is a need of more information on acid decontamination at those lower temperatures.

In a study by Berk *et al.* (2005), significant strain variability regarding acid resistance was demonstrated [10], however potential differences between *Salmonella* isolates from feed seems not to have been studied. Some authors have raised concern that the use of organic acids for decontamination of animal feed might result in selection or emergence of acid-tolerant strains that are more likely to survive gastric acidity as well as to develop tolerance to other stress factors such as heat, osmosis and salts [11-14]. Berk *et al.* (2005) pointed out the correlation between acid resistance and pathogenicity [10]. For this reason, it is of importance that acid treatment for decontamination of *Salmonella* must be effective even against less susceptible strains.

Clearly, there are several factors and conditions that affect the efficacy of acids for decontamination of *Salmonella*. As pointed out by Wales *et al.* (2010) variation in results may in addition be due to variation in the methodology and media used for isolating and enumerating *Salmonella*, technical aspects such as method of application, mechanical failures, sedimentation effects or miscalculations of dose rates [4].

In order to improve recommendations for the stakeholders about the efficacy of organic acids for decontamination of *Salmonella* in feed more information is needed. The aims of the present study were to investigate: a) the efficacy of FA, PA and commercial mixtures of organic acids for reduction in *Salmonella* in feed materials, b) the variation in acid tolerance among *Salmonella* strains isolated from feed, c) whether lower temperatures reduced the decontamination by acids compared to room temperature.

## Results

### Selection of strains

The *Salmonella* strains used in this study (*Salmonella* ser. Typhimurium 98/1991, *Salmonella* ser. Senftenberg 252/1995, *Salmonella* ser. Infantis 167/2007, *Salmonella* ser. Putten 297/2007) were selected on the basis of differences in acid tolerance. Two strains were selected because of a high acid tolerance (*S.* Putten and *S.* Infantis) and two strains because of a low acid tolerance (*S.* Typhimurium, *S.* Senftenberg). The *S.* Typhimurium and *S.* Senftenberg strains were isolated in 1991 and 1995, respectively because acid treatment was not used for raw materials at that time. The *S.* Infantis and *S.* Putten strains were both isolated in 2007 i.e. after acid treatments of contaminated ingredients became common practise. The strains were selected out of nine initial strains and ranked according to their average acid tolerance defined as the sum of the calculated area under the survival curve (AUC) in culture broth. In the following order (highest to lowest tolerance): *S.* Putten 297/2007, *S.* Infantis 167/2007, *S.* Agona 23/1992, *S.* Livingstone 860/1993, *S.* Putten 355/2007, *S.* Senftenberg 252/1995, *S.* Typhimurium 98/1991, *S.* Emek 782/2007 and *S.* Reading 655/2007.

### Trial 1 and 2. Survival analysis of *Salmonella* with and without organic acids

**Figure 1:**
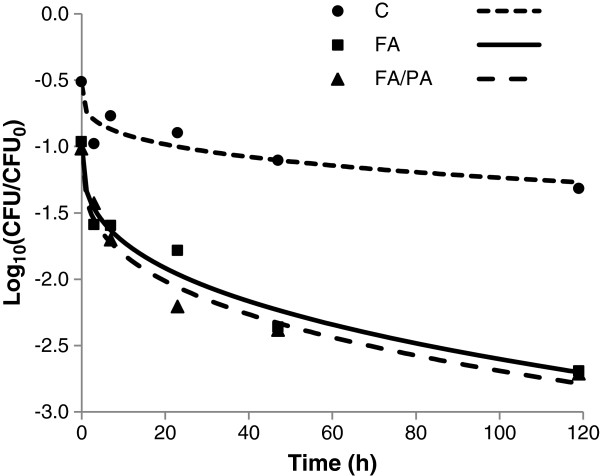
**Effect of organic acids on the survival of *Salmonella *in rape seed meal.** Recovery of the *S.* Typhimurium strain and the *S.* Infantis strain in rapeseed meal at different time points without exposure to acid (control) or exposed to 1% formic acid (FA) or a 1% mixture of formic and propionic acid (FA/PA) (80%:20%). The points show the mean values for two separate experiments with three replicate samples for each of the *Salmonella* strains. The lines show the fitted Weibull models with the following parameters C: N_0_=−0.53 log_10_ CFU/ml (SE 0.14), Δ= 359 h (SE 357 h), p=0.28 (SE 0.14). FA: N_0_=−0.98 log10 CFU/ml (SE = 0.14); Δ=24.40 h (SE 12.4 h), p= 0.34 (SE 0.08). FA/PA: N_0_=−0.98 log_10_ CFU/ml (SE 0.14), Δ=18 h (SE 8.5), p=0.31 (SE 0.12).

In trial 1, after 1 h treatment with 1% FA or 1% FA/PA, an approximate 0.5 log_10_ reduction in *Salmonella* was obtained compared with the control (Figure 1). After 48 h an additional 0.5 log_10_ reduction in *Salmonella* was obtained and after 120 h the reduction was approximately 1.5 log_10_ greater compared with the control. The results did not indicate significant differences between treatment with FA or FA/PA (Figure 1). The survival curves after acid exposure showed a concave shape, with a rapid initial death phase followed by a slower rate of reduction. The shape of the survival curve was used to select time-points for further trials.

**Figure 2:**
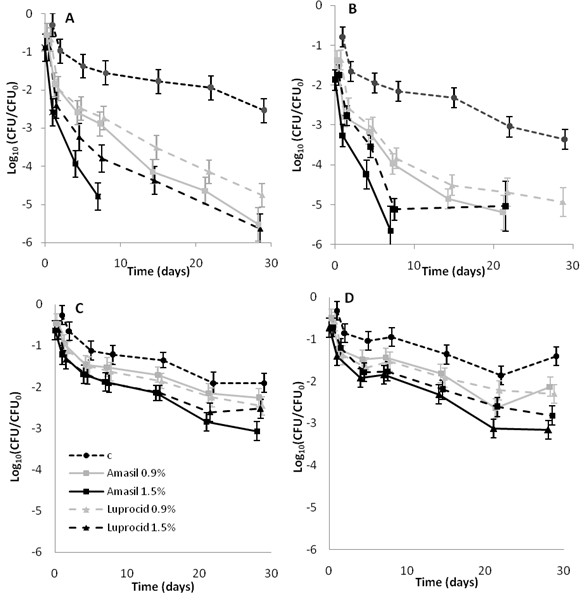
**Effect of commercial blends of acids on the survival of *Salmonella *in feed materials.** Recovery of *Salmonella* in acid treated feed materials. Compound mash feed (**A**, **B**) and soybean meal (**C**, **D**) were pre-treated with 0.9 or 1.5% of Amasil or Luprocid respectively, and inoculated with the *S.* Infantis strain (**A**, **C**) or the *S.* Typhimurium strain (**B**, **D**). Samples were collected at 0, 1, 4, 7, 14, 21 and 28 days plated on TSA followed by XLD overlay (c = control).

In trial 2, we observed a difference in *Salmonella* survival between the feed types, with a more profound effect in compound mash feed compared to soybean meal (Figure 2). At most time points the reduction in *Salmonella* was approximately 0.5-1 log_10_ higher with Amasil compared with Luprocid in compound mash feed (Figure 2A, B). In soybean meal a difference between Amasil and Luprocid could be seen after 14 days at 1.5% concentration. A statistically significant difference in time-dependent reduction was seen between the strains (conc*strain*time p=0.02). In both materials, a significant effect of acid concentration was observed (Figure 2). Also in this trial the survival curves showed a concave shape, with a fast initial reduction followed by reduction at a slow rate.

### Trial 3. Effect of strain, acid type and feed material in a multifactorial experiment

**Figure 3:**
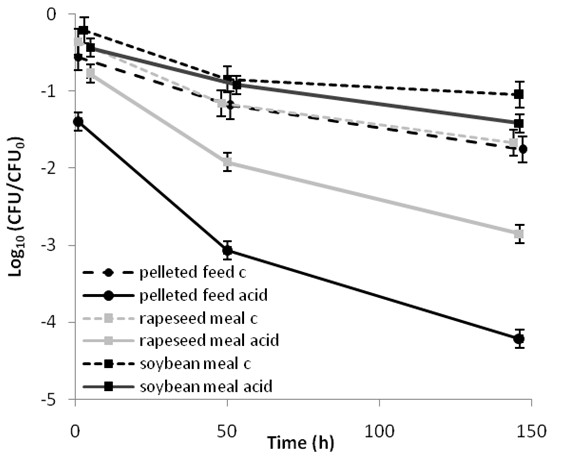
**Effect of strain, acid type and feed material on the survival of *Salmonella*.** Recovery of the *S.* Senftenberg, *S.* Typhimurium, *S.* Infantis and *S.* Putten strains in different feed materials at different time points without (dashed lines; c = control) or with (solid lines) exposure to 1% formic acid or a mixture of formic and propionic acid (80%:20%) following 1, 48 or 144 h incubation.

The reduction in *Salmonella* after acid treatment was greater in pelleted feed compared with rape seed meal, whereas only a minor reduction was seen in soybean meal (Figure 3). In agreement with the results from trial 1 the ANOVA did not indicate any differences between results obtained with 1% FA compared with 1% FA/PA in any feed material or at any time point. Therefore, in further calculations the two acid treatments were considered equivalent.

The statistical analysis showed that all factors (strain, acid, feed material and time) significantly influenced the rate of reduction in *Salmonella.* The analysis of cross terms showed that in the presence and absence of acids the feed material had a significant effect on the time-independent (material, material*acid) and the time-dependent (material*time, material*acid*time) reduction in *Salmonella*.

In order to quantify the effects of acid treatment in different feed materials, the ANOVA was repeated with only the significant variables and predicted values for the recovery (CFU/CFU_0_) were calculated for each combination of time, acid and material (Figure 3). In the three feed materials, acid treatment resulted in statistically significant (p<0.05) reduction in *Salmonella* recovery after only 1 h of exposure and the difference in reduction between control and acid treated samples increased with time (Figure 3). In soybean meal, the difference in *Salmonella* reduction between the acid treated samples and the controls was less than 0.5 log_10_ at 144 h. In rapeseed meal and pelleted feed the difference in reduction between acid treated samples and controls at 144h was approximately 1 log_10_ and 2.5 log_10_, respectively (Figure 3).

In trial 3, the strains were ranked with respect to acid tolerance by comparing the calculated area under the survival curve (AUC). In pelleted compound feed *S.* Infantis was found to be the most acid tolerant strain followed by the *S.* Putten*, S.* Senftenberg and *S.* Typhimurium strains*.* Except for the relative order of the *S.* Putten strain and the *S.* Infantis strain, the ranking of the four strains in this study was similar to the results obtained in the pilot study where the nine different *Salmonella* strains were screened for their differences in acid tolerance. However, the differences were not statistically significant in the ANOVA due to the small number of replicates*.* Minor differences between strains could be detected in soybean or rape seed meal (data not shown).

### Trial 4. Reduction in *Salmonella* due to formic acid in pelleted feed

To verify the difference in acid tolerance between strains additional replicates were performed with the *S.* Typhimurium and *S.* Infantis strains. The trial was restricted to pelleted compound feed since the previous results indicated that experimental variation would overrule any effect of strain in the other materials.

**Figure 4:**
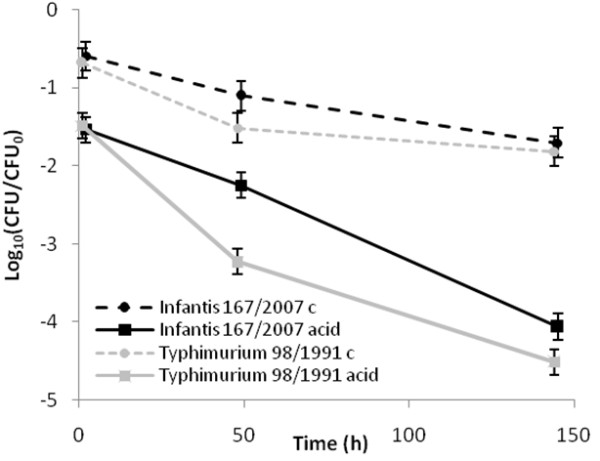
**Differences between *Salmonella *strains regarding acid tolerance in pelleted feed.** Recovery of the *S.* Infantis strain or the *S.* Typhimurium strain in pelleted feed with or without exposure to 1% acid following 1, 48 or 144 h incubation. The data points are based on the combined data from samples treated with formic acid or a mixture of formic and propionic acid (80%:20%) in trials 3 and 4.

After 48 h, the difference in reduction between the strains was approximately 1 log_10_ but a difference in reduction between strains was also seen in the control. The difference between acid treatment and control was approximately 0.5 log_10_ higher for the *S.* Typhimurium strain than for the *S.* Infantis strain (Figure 4). After 144 h of acid treatment, the recovery of the *S.* Typhimurium strain was approximately 0.5 log_10_ lower than for the *S.* Infantis strain (Figure 4). ANOVA on the combined data (trial 3 and 4) showed that the choice of strain had a significant effect on the observed reduction in *Salmonella* (acid*strain p=0.02; acid*strain*time p=0.02).

### Trial 5. Effects of formic acid at temperatures below room temperature

**Figure 5:**
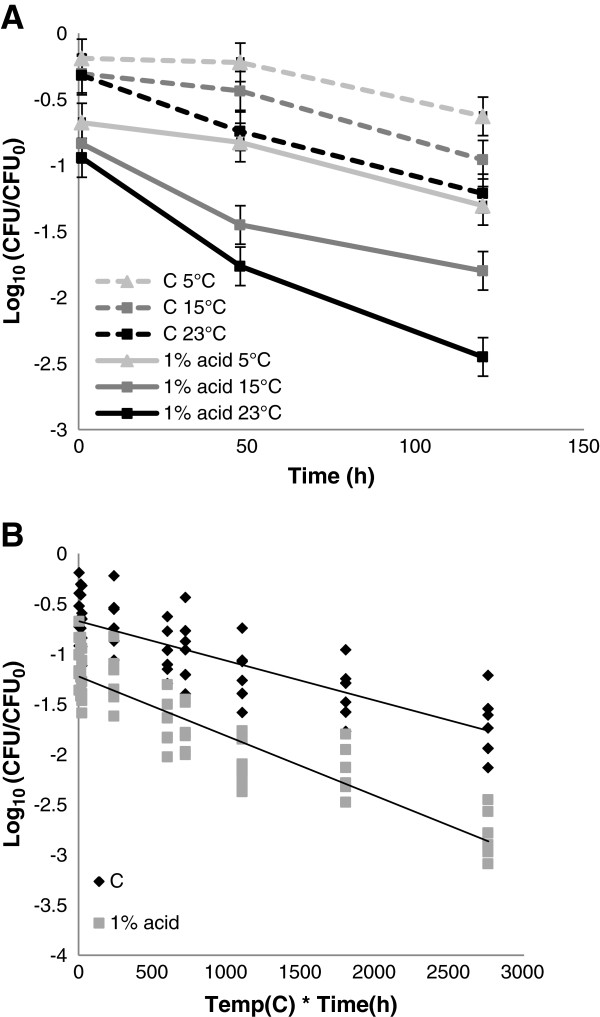
**Effect of temperature on acid induced reduction in *Salmonella*.** (**A**) Recovery of the *S.* Typhimurium strain and the *S.* Infantis strain in rapeseed meal with or without exposure to 1% formic acid at 5°C, 15°C and room temperature (~23°C) after 1, 48 and 120h (c = control). (**B**) Reduction in *Salmonella* in feed materials as function of accumulated temperature (temp*time). Data are pooled for rape seed meal and soybean meal.

In rape seed meal (Figure 5A) and in soybean meal (results not shown) a significant effect of temperature on the reduction of *Salmonella* was observed. At 5°C and 15°C a longer incubation time was necessary to obtain the same level of acid dependent reduction in *Salmonella* compared with 23°C. Also in control samples the reduction was lower at 5°C and 15°C compared to 23°C. No statistically significant interaction between the factors “material” and “temperature” was observed. A significant correlation between the accumulated temperature (temp*time) and the reduction in *Salmonella* was observed (Figure 5B). The correlation was stronger in presence of acid (r^2^ = 0.83, p=0.04) compared to the control (r^2^ = 0.67, p= 0.07).

## Discussion

In the present study, an overlay method was used to enhance the recovery of injured *Salmonella* cells. It is a well-established technique that has been shown to increase the recovery of bacterial cells injured by various treatments including acidification [15-17].

The actual numbers of *Salmonella* cells added to the feed materials were not recovered in the control samples. One explanation might be that some cells were injured, e.g. due to dehydration, leading to non-culturable cells despite a sensitive isolation method used. Another explanation might be that the organic acids masked the presence of *Salmonella* when analysed with culture-based methods [18]. In this study differences in the survival of *Salmonella* between acid treated feed materials and controls and increased with time, indicating that the effects observed were not an artefact of the detection method, but reflected a true reduction in viable *Salmonella* cells.

The initial survival analysis in trial 1 was performed in rape seed meal, since pilot studies indicated that the effect of acid in this commodity was intermediate. The results showed that 1 h after addition of acid, there was a decrease in *Salmonella* counts compared with the control (Figure 1). However, the immediate acid effect is difficult to interpret, since masking effects, imperfect recovery and sublethally injured *Salmonella* on the TSA plates might have contributed to these results.

Significant differences in the effects of acids on reduction of *Salmonella* between different feed materials were observed (Figure 3). The reasons why the efficacy of acid treatment was poorer in some feed materials is currently unknown. The fact that similar results were obtained with pelleted and compound mash feed suggests that the differences between feed materials were not due to surface area or structure, but rather to differences in chemical composition. It has been demonstrated that protein-rich and/or fat-rich matrices have a protective effect on *Salmonella* cells, e.g. by enhancing the buffering capacity [19] or by conversion of PA to its less active form by protein ingredients in the feed [20]. In agreement with this, the slowest reduction in *Salmonella* was seen in soybean meal with the highest protein content (47.8%) and the fastest reduction in compound feed with the lowest protein content (15.5%).

Since batches with sufficiently high counts of *Salmonella* are rare, almost all studies including the present are based on artificially contaminated materials. However, the reduction in *Salmonella* observed in the present study is in the same magnitude as a technical report by Hansen *et al.* (1995) where naturally contaminated materials were treated with 1% FA [21]. The agreement with the results from the present study and from Hansen *et al.* indicates that the model system can to some degree predict reduction of *Salmonella* in naturally contaminated feed materials. The authors pointed out that it was not possible to judge whether the differences in *Salmonella* reduction in cottonseed and rapeseed expellers could be attributed to differences between feed materials, strain characteristics or batch history. In the present study significant differences between strains were observed although the differences were not as large as the differences observed between materials. The screening of *Salmonella* strains for acid resistance showed that the strains isolated in 1991 and 1995 compared to 2007 did not differ in acid tolerance. However, as the numbers of strains were limited, it cannot be ruled out that acid tolerant *Salmonella* has increased since the introduction of acid treatment in the feed chain.

*Salmonella* counts in naturally contaminated feed or feed ingredients are usually low [22,23]. With the observed reduction in *Salmonella* counts in compound feed the levels after acid treatment would probably be less than 1 CFU/kg. This suggests that the risk of *Salmonella* being introduced to farms through acid-treated compound feed is small. With the low initial counts of *Salmonella* a moderate reduction seen in rape seed and soybean meal is likely to result in *Salmonella* counts below detection limit and there is a high probability that the material would be tested negative following sampling. However, even low numbers of surviving *Salmonella* per kg could still cause problems if conditions favouring growth would occur later in the processing line or in storage areas [2].

Commercial blends of acids are commonly used by the feed industry despite limited information from the suppliers. For that reason a study with Amasil and Luprocid was carried out. Many commercial suppliers of acid products propose a 48h treatment of contaminated feed materials. In this study, the reduction in *Salmonella* was monitored over 4 weeks since the earlier studies showed that the reduction in soybean meal was limited after 7 days. A prolonged incubation time resulted in an additional reduction in *Salmonella* counts, although the acid effect in soybean meal was poor in comparison with the un-treated meal. Amasil was slightly more efficacious than Luprocid. Considering that the concentration of FA is lower in Amasil than in Luprocid, SF might have an additional effect on *Salmonella* in feed, which might be distinct from the pH reducing effect. From an industrial perspective the use of acid/salt blends such as Amasil might be advantageous, since pure acids are corrosive to the equipment.

Since the temperature in the greater part of the year in the Nordic countries is low we were interested to study effects at lower temperatures. Cherington *et al.* (1992) showed that the bactericidal effect of organic acids in culture broth decreased with lower temperatures. Results from the present study using rape seed and soybean meal shows that application of FA at lower temperatures result in poorer decontaminating effects compared to room temperature (Figure 5). This effect was expected since biological and chemical reactions slow down at lower temperatures and should be considered in recommendations from commercial suppliers. Acid treatment at lower temperatures would require longer incubation time in order to obtain the desired reduction level compared to room temperature.

The results indicate that the recommended incubation time for acid treatment of feed materials should be based on accumulated temperature (degree hours) since this gives a better prediction of the rate of acid dependent reduction at lower temperatures.

## Conclusions

The efficacy of different acids on reduction of *Salmonella* varied significantly between feed materials. Although a prolonged incubation time was applied, the effect in soybean meal was poor. Equivalent efficacy results were obtained for FA and a blend of FA and PA, whereas the commercial product Amasil (blend of FA and SF) was slightly more efficacious than Luprocid (blend of FA and PA) in compound mash feed. The investigated strains showed a variation in acid tolerance and at lower temperatures reduced effects of acids were seen compared to room temperature.

In this study, we could confirm previous results that acid treatment of *Salmonella* in feed is a matter of reducing the number of viable bacterial cells rather than eliminating the organism. Many commercial suppliers of acid products propose a 48 h treatment of *Salmonella* contaminated feed materials. Based on these results recommendations on the use of acids for controlling *Salmonella* in feed should take into account the relative efficacy of acid treatment in different feed materials, the variation in acid tolerance between different *Salmonella* strains, and the treatment temperature. If ‘*Salmonella*-free’ feed is the aim it is essential to combine acid treatment with other decontamination procedures.

## Methods

### Feed materials

Pelleted compound pig feed (15.5% protein and 2.2% fat) (see Additional file 1), extracted soybean meal (47.8% protein and 0.8% fat) and rape seed meal (34.1% protein and 2.6% fat) were used in the experiments with organic acids.

In the experiments with commercial blends of acids, a compound mash feed for fattening pigs, in a composition typical for European countries, (see Additional file 2) and soybean meal were used. The two feed types were produced with or without Amasil (61% formic acid, 20.5% sodium formate, 18.5% water) and Luprocid (75% formic acid, 25% propionic acid), added at levels of 0.9 and 1.5%, respectively. Amasil and Luprocid were from BASF, Lampertheim, Germany. The mash feed and soybean meal was produced in a 250 kg batch. The two commercial acid mixtures were added to subsamples of 10 kg and mixed in a high precision mixer. Subsamples from the acid treated feed were then used in trial 2.

The reason why different types of feed were used in some of the trials was due to the involvement of different laboratories and that the studies were carried out during different time periods.

Before the experiments started samples of all feed materials were analysed with the NMKL71 method for any pre-existing *Salmonella* contamination.

### *Salmonella* strains and preparation of inoculum

The following strains of *Salmonella enterica* ssp. *enterica* isolated from feed, from the culture collection at the National Veterinary Institute, Uppsala, Sweden, were used in the study (strain number/isolation year): *Salmonella* ser. Typhimurium 98/1991, *Salmonella* ser. Senftenberg 252/1995, *Salmonella* ser. Infantis 167/2007, *Salmonella* ser. Putten 297/2007.

These four strains were selected out of nine *Salmonella* strains, previously investigated and compared in a pilot study for their acid tolerance for FA or FA/PA in culture broth and in presence or absence of 20% NaCl (data not shown).

Stock cultures were maintained in Tryptic Soy Broth (TSB) (Oxoid CM0129, Basingstoke, Hampshire, England) with 50% glycerol at −20°C. In order to activate the strains, 100 μl from the stock cultures were transferred to 10 ml TSB and incubated at 37 ± 1°C for 24 h. Then 100 μl were transferred to fresh 10 ml TSB and incubated at 37 ± 1°C for 18 h. The cultures were harvested by centrifugation (Labofuge 400R, Heraeus Instruments, Osterode, Germany) at 3500 rpm for 10 min at 4°C and washed twice in 10 ml peptone saline water (PSW) in the experiments with pure organic acids, and PBS in the experiments with commercial blends of acids. The cells were resuspended in 9 ml fresh PSW to be used immediately as inoculum in the experiments at a concentration of 10^8^ to 10^9^ CFU/ml (estimated using plate count). If necessary, the cell suspensions were diluted in a 10-fold dilution series.

### Trial 1 and 2. Survival analysis of *Salmonella* with and without organic acids

The effects of organic acids or commercial blends of acids on the survival of *Salmonella* in feed materials were investigated in two separate trials. All acids and blends of acids were in liquid form.

In trial 1 organic acids were used in order to study the kinetics of acid reduction and to select appropriate time points for further trials. The study was conducted in three replicates, where 0.25 ml inoculum of the *S.* Typhimurium strain or the *S.* Infantis strain, respectively, were added by pipette to 10 g of rape seed meal and homogenized thoroughly by vortexing or using a spatula. The samples were then exposed to 1% FA or 1% mixture of FA and PA (80%:20%) added by pipette and homogenized thoroughly by vortexing or using a spatula. Non-acid treated samples were used as controls. After incubation for 1, 4, 8, 24, 48 and 120 h at room temperature (20°C ± 2°C) the samples were incubated for 30 min in 90 ml buffered peptone water (BPW) (Oxoid CM 0509, Basingstoke, England). Then, a 1 ml aliquot was diluted in a 10-fold dilution series in PSW. Two times 100 μl of each culture were then plated on duplicate TSA-agar plates (Difco, no. 236950, Le Pont de Claix, France) with 0.1% sodium pyruvate. The plates were incubated at 37 ± 1°C for 2 h followed by overlaying with 10 ml xylose lysine deoxycholate (XLD) agar (0.0015% novobiocin; Lab M lab 32, Axel Johnson Lab System Inc., Solna, Sweden) and colonies were manually counted after incubation at 37 ± 1°C for 22 h ± 2h. Colonies showing a typical appearance were counted. The experiment was repeated once.

The plate counts were used to calculate the concentration of organisms (CFU/ml) in the feed mixed with BPW or PBS. The calculation was based on plates with <300 colonies where:

$$c=10\times \frac{\sum_{i=1}^{n}\mathrm{ni}}{\sum_{i=1}^{n}d_{i}}$$

c is concentration in CFU/ml, n_i_ is number of colonies on plate i and d_i_ is the dilution factor of plate i. The values obtained were normalised against the concentration with which the feed materials were inoculated by dividing the value per ml obtained (CFU) by the artificial contamination concentration (CFU_0_). The results obtained from the plate counts were transformed into log_10_ values and log_10_ reduction was calculated as log_10_ ((CFU/ml recovered)/ (CFU/ml added)).

In trial 2 two commercial acid products were used. The study was conducted in 4 g of compound mash feed and soybean meal with 0.9 or 1.5% Amasil, or with 0.9 or 1.5% Luprocid, prior to artificial contamination. The same *Salmonella* strains and procedure as in trial 1 was used except that 0.1 ml from an appropriate dilution was used. The inoculated feed samples were stored at room temperature (20°C ± 2°C) for 0, 1, 4, 7, 14 and 28 days. Then 36 ml of PBS were added to the feed samples, mixed thoroughly and a 1 ml aliquot was diluted in a 10-fold dilution series in PBS. The same procedure as in trial 1 for plating and plate count was used. The manufacturer in this case of TSA and XLD was Oxoid, no. BO0330V (with 0.1% sodium pyruvate) and no. CM0469B (Basingstoke, Hampshire, England), respectively. Two separate experiments were performed in duplicate for each feed type and duplicate measurements were performed on each sample.

### Trial 3. Effect of strain, acid type and feed material in a multifactorial experiment

*Salmonella* survival was investigated after 1, 48 and 144 h. Two separate experiments were performed using the same procedure as in trial 1 except that 0,5 ml of the inoculums were used. *Salmonella* and acid was added to pelleted compound pig feed by distributing as evenly as possible by pipette followed by vortexing without homogenization by spatula. For each time point one sample from every combination of strain (*S.* Typhimurium, *S.* Senftenberg, *S.* Infantis and *S.* Putten), material (rape seed meal, soybean meal, pelleted feed) and acid mix (1% FA vs. 1% FA/PA (80%:20%) was analysed.

### Trial 4. Reduction in *Salmonella* due to formic acid in pelleted feed

The acid resistance of the *S.* Typhimurium strain and the *S.* Infantis strain was investigated in pelleted compound feed with 1% FA. This experiment was performed once in quadruplicate, with measurements after 1, 48 and 144 h. The same procedure as in trial 1 was used except that 0,5 ml of the inoculums were used.

### Trial 5. Effects of formic acid at temperatures below room temperature

The influence of different temperatures (5°C ± 1°C and 15°C ± 1°C) compared to room temperature (23°C ± 1°C) on the effect of 1% FA on *Salmonella* in rape seed meal and soybean meal was investigated. Two separate experiments using the *S.* Typhimurium strain and the *S.* Infantis strain were performed where each treatment was applied to single samples with duplicate measurements after 1 h, 48 h and 120 h. The same procedure as in trial 1 was used.

### Statistical analysis

#### Analysis of survival curves

The survival curves were fitted by a Weibull model using GinaFit 1.5 for excel 2007 downloaded from (http://cit.kuleuven.be/biotec/downloads.php) accessed March 1^st^ 2011.

#### Analysis of variance and post estimation

The dataset was analysed for outliers and missing data points before analysis of variance by ANOVA. Two data points were excluded from the datasets.

The results were analysed by one-way ANOVA using Stata/IC 11.1 for Windows (StataCorp LP, College Station, Texas, USA). The ANOVA was followed by regression (function *regress*) and estimation of the predicted value ŷ_i_ and standard deviation in prediction *s*_ŷ_ for each observation (function *predict*). The length of error bars is the confidence interval for the prediction calculated as: ŷ_i_ +/− 1.97* *s*_ŷ_ .

The following variables were used:

Replicate (1, 2)

Log (recover) = log_10_(CFU_recovered_/CFU_added_)

Strain (1 = the *S.* Infantis strain, 2 = the *S.* Putten strain, 3 = the *S.* Senftenberg strain, 4 = the *S.* Typhimurium strain)

Acid type (0 = control, 1 = FA, 2 = FA/PA) (trial 1)

Concentration (0%, 0.9%, 1.5%) (trial 2)

Acid (0 = control 1 = FA or FA/PA) (trial 3–5)

Time (1, 48, 144 h)

Material (1 = soybean meal, 2 = rapeseed meal, 3 = pelleted feed)

Temperature (5°C, 15°C, 23°C) (trial 5 only)

For trial 3, the significance of each variable and their interactions were analysed using the model: Log (recover) = strain material acidtype time replicate acidtype*time acidtype*strain acidtype*material acidtype*strain*time acidtype*material*time acidtype*strain*material acidtype*strain*material*time. Since no differences were observed between the acid types, the variable acidtype was replaced by the variable acid. Predicted values and confidence intervals for *Salmonella* reduction in different feed materials (trial 3) were calculated using the variables material, acid and time. To analyse the difference in acid tolerance between the *S.* Typhimurium strain and the *S.* Infantis strain, data from trial 3 and 4 were analysed as above using the variables acid, strain and time.

Each data point in the figures represents the predicted value ŷ_i_ for log_10_ (recovered CFU/CFU_0_) for the time and treatment based on the regression analysis, where CFU_0_ is the amount of *Salmonella* used for artificial contamination. The error bars indicate the confidence interval for the predicted value. Data points in the diagram are shifted along the x-axis to avoid points and error bars from different data series being superimposed.

#### Regression analysis

The correlation between accumulated temperature and *Salmonella* reduction was analysed in Microsoft Office Excel 2007, function REGR().

## Competing interests

The authors declare that they have no competing interests.

## Authors’ contributions

SK took part in the design of the study, performed the laboratory procedures, participated in the analysis and interpretation of data and drafted the manuscript. GA performed the statistical analysis and helped to draft the manuscript as well as took part in the design of the study. CL participated in the analysis and interpretation of data and helped to review the manuscript. PNS participated in the statistical analysis and reviewed the manuscript. AG participated in the design and pilot experiments. JZ participated in the design of the study and reviewed the manuscript. PH coordinated the study and reviewed the manuscript. All authors read and approved the final manuscript.

## Supplementary Material

Additional file 1 Ingredient and nutrient composition of the pelleted compound feed intended for growing pigs. Click here for file

Additional file 2 Ingredient and nutrient composition of mashed compound feed (basal starter diet intended for pigs). Click here for file
