# Supplementary material for: Organic acids for control of Salmonella in different feed materials
Source: BMC Vet Res. 2013 Apr 18;9:81. doi: 10.1186/1746-6148-9-81 (PMC3646707; doi:10.1186/1746-6148-9-81)
Supplement: Additional file 1 — Ingredient and nutrient composition of the pelleted compound feed intended for growing pigs. [file 1746-6148-9-81-S1.docx]

Additional file 1. Ingredient and nutrient composition of the pelleted compound feed intended for growing pigs

| **Basal diet** |  |  |
| --- | --- | --- |
| **Ingredients:** |  |  |
| Barley | g/kg | 165.00 |
| Wheat | g/kg | 417.00 |
| Oats  Peas  Soybean meal | g/kg  g/kg  g/kg | 20.00  125.00  7.50 |
| Calcium carbonate | g/kg | 15.00 |
| Rape seed meal (extracted)  Sugar beet molasses | g/kg  g/kg | 73.00  15.00 |
| Vitamin-mineral premix* | g/kg | 12.00 |
| Monocalcium phosphate | g/kg | 4.00 |
| Wheat feed meal | g/kg | 80.00 |
| Sodium chloride | g/kg | 5.00 |
| Wheat bran | g/kg | 50.00 |
| Triticale | g/kg | 20.00 |
| **Composition:** |  |  |
| Metabolizable Energy_pigs_ | MJ/kg | 12.4 |
| Crude protein | g/kg | 150.00 |
| Crude fibre | g/kg | 53.00 |
| Crude fat | g/kg | 29.00 |
| Lysine | g/kg | 8.30 |
| Methionine | g/kg | 2.50 |
| Threonine | g/kg | 5.20 |
| Potassium | g/kg | 7.00 |
| Methionine + Cystine | g/kg | 5.60 |
| Calcium | g/kg | 8.00 |
| Phosphorus | g/kg | 5.00 |
| Sodium | g/kg | 2.40 |
| Nitrogen | g/kg | 24.00 |
| Crude ash | g/kg | 53.00 |
| Water | g/kg | 123.00 |

* Premix Contents per kg : Vit. A 4000 IE ; Vit. D_3_ 400 IE ; Vit. E 60 mg ; Cu (Copper sulfate); 15 mg Se (Sodium selenite); 0.4 mg
