# Supplementary material for: Organic acids for control of Salmonella in different feed materials
Source: BMC Vet Res. 2013 Apr 18;9:81. doi: 10.1186/1746-6148-9-81 (PMC3646707; doi:10.1186/1746-6148-9-81)
Supplement: Additional file 2 — Ingredient and nutrient composition of mashed compound feed (basal starter diet intended for pigs). [file 1746-6148-9-81-S2.docx]

Additional file 2. Ingredient and nutrient composition of mashed compound feed (basal starter diet intended for pigs)

| **Basal diet** |  |  |
| --- | --- | --- |
| **Ingredients:** |  |  |
| Barley | g/kg | 498.40 |
| Wheat | g/kg | 279.00 |
| Soybean meal (44% CP) | g/kg | 174.00 |
| Calcium carbonate | g/kg | 13.50 |
| Soy oil | g/kg | 12.00 |
| Vitamin-mineral premix* | g/kg | 12.00 |
| Monocalcium phosphate | g/kg | 7.00 |
| L-Lysine | g/kg | 2.00 |
| Sodium chloride | g/kg | 1.60 |
| L-Threonine | g/kg | 0.30 |
| DL-Methionine | g/kg | 0.20 |
| **Composition:** |  |  |
| Metabolizable Energy_pigs_ | MJ/kg | 12.79 |
| Crude protein | g/kg | 165.10 |
| Crude fibre | g/kg | 47.50 |
| Crude fat | g/kg | 29.60 |
| Lysine | g/kg | 9.40 |
| Methionine | g/kg | 2.80 |
| Threonine | g/kg | 6.30 |
| Tryptophan | g/kg | 2.50 |
| Methionine + Cystine | g/kg | 5.90 |
| Calcium | g/kg | 7.20 |
| Phosphorus | g/kg | 5.40 |
| Sodium | g/kg | 1.80 |

* Premix Contents per kg Premix: 400000 U Vit. A; 40000 U Vit. D_3_; 8000 mg Vit. E (α-Tocopherole acetate); 300 mg Vit. K_3_; 250
 mg Vit. B_1 ;_ 250 mg Vit. B_2_; 2500 mg Nicotinic acid; 400 mg Vit. B_6_; 2000 μg Vit. B_12_; 25000 μg Biotin; 1000 mg
 calcium pantothenate acid; 100 mg Folic acid; 80000 mg Choline chloride; 5000 mg Zn (Zinc oxide); 2000 mg Fe
 (Iron carbonate); 6000 mg Mn (Manganese oxide); 1200 mg Cu (Copper sulfate-pentahydrate); 45 mg J (Calcium jodate);
 30 mg Co (Cobalt- (II)-sulfate-heptahydrate); 35 mg Se (Sodium selenite); 130 g Na (Sodium chloride); 55 g Mg
 (Magnesium oxide).
